# Genome-wide association study identifies a locus associated with rotator cuff injury

**DOI:** 10.1371/journal.pone.0189317

**Published:** 2017-12-11

**Authors:** Thomas R. Roos, Andrew K. Roos, Andrew L. Avins, Marwa A. Ahmed, John P. Kleimeyer, Michael Fredericson, John P. A. Ioannidis, Jason L. Dragoo, Stuart K. Kim

**Affiliations:** 1 Department of Developmental Biology, Stanford University Medical Center, Stanford, CA, United States of America; 2 Department of Health Research and Policy, Division of Epidemiology, Stanford University Medical Center, Stanford, CA, United States of America; 3 Kaiser Permanente Northern California, Division of Research, Oakland, CA, United States of America; 4 Department of Physical Medicine and Rehabilitation, Harvard Medical School, Boston, MA, United States of America; 5 Department of Orthopaedic Surgery, Stanford University Medical Center, Stanford, CA, United States of America; 6 Department of Medicine, Stanford Prevention Research Center and Dept. of Health Research and Policy, Division of Epidemiology, Stanford University School of Medicine, and Dept. of Statistics, Stanford University School of Humanities and Sciences, Stanford, CA, United States of America; University of Northampton, UNITED KINGDOM

## Abstract

Rotator cuff tears are common, especially in the fifth and sixth decades of life, but can also occur in the competitive athlete. Genetic differences may contribute to overall injury risk. Identifying genetic loci associated with rotator cuff injury could shed light on the etiology of this injury. We performed a genome-wide association screen using publically available data from the Research Program in Genes, Environment and Health including 8,357 cases of rotator cuff injury and 94,622 controls. We found rs71404070 to show a genome-wide significant association with rotator cuff injury with p = 2.31x10^-8^ and an odds ratio of 1.25 per allele. This SNP is located next to *cadherin8*, which encodes a protein involved in cell adhesion. We also attempted to validate previous gene association studies that had reported a total of 18 SNPs showing a significant association with rotator cuff injury. However, none of the 18 SNPs were validated in our dataset. rs71404070 may be informative in explaining why some individuals are more susceptible to rotator cuff injury than others.

## Introduction

Rotator cuff injuries are a common cause of shoulder pain throughout life. The prevalence of rotator cuff tears in the general population is approximately 20%; increasing from 10% in the sixth decade of life to 50% in the ninth decade of life[1]. While the aetiology of rotator cuff tears is poorly understood, the literature supports an age-related progression, primarily affecting middle-aged and older patients. In addition to aging, other risk factors for degenerative tears include smoking, hypercholesterolemia, genetic predisposition and shoulder use[2].

40% of overhead throwing athletes were found to have rotator cuff tears in their dominant arm[3]. Athletes participating in overhead sports place substantial demands on the shoulder–from elite swimmers ranging their shoulders through approximately 2 million strokes per year to professional baseball pitchers generating ball speeds of up to 165 km/hr with associated peak internal rotation velocities of up to 6940 degrees per second[4]. Given these high demands, rotator cuff injury can have devastating consequences on an athlete’s performance, require substantial recovery and rehabilitation time, or even prematurely end an athlete’s career[5,6].

Multiple lines of evidence suggest that genetic differences explain part of the predisposition for rotator cuff injury. First, siblings and other close relatives have an elevated risk of rotator cuff tears and show a faster rate of progression of tear size compared to non-related controls[7,8]. Second, genetic association studies have previously identified 18 single nucleotide polymorphisms (SNPs) associated with rotator cuff tears[9–14]. From a cohort with 335 cases, Tashjian et al. identified two genome-wide significant SNPs that showed an association with full thickness rotator cuff tears[12]. Moreover, several candidate gene studies have been performed to identify 16 additional SNPs associated with rotator cuff tears[9–11,13,14]. For rotator cuff injury, none of these 18 SNPs have yet been validated in an independent study. One SNP in the ESRRB gene remained associated with rotator cuff injury when the cohort was expanded from 175 to 335 cases[12,13].

A better understanding of the genetic loci involved in rotator cuff injury could shed light on the poorly understood molecular basis of tendon injury and repair. Although an individual’s genotype is fixed, knowledge of this risk could prompt dedicated preventative measures to potentially decrease the risk of injury.

The purpose of this genome-wide association study (GWAS) was to identify SNPs associated with rotator cuff injury using available data from a cohort of 102,979 patients that included 8,357 cases with rotator cuff injury.

## Materials and methods

A genome-wide association screen was performed for rotator cuff injury using data from the Genetic Epidemiology Research on Adult Health and Aging (GERA) cohort. The data generation and data analysis pipeline have been previously described in Jorgenson et al 2015[15]. Supplemental Methods contains full information about the methods used.

### Phenotype definition

Rotator cuff injury cases were identified in the GERA cohort based on clinical diagnoses and surgical procedures captured in the Kaiser Permanente Northern California (KPNC) Electronic Health Record system. The Electronic Health Record includes reported injuries over the entire lifetime of the patients, including those that occurred prior to enrollment in KPNC. It also includes injuries that occurred after the genotyping analysis was performed if reported by the patient and recorded by the physician, until the data were accessed on July 22, 2015. Nine total codes, including International Classification of Disease, Ninth Revision (ICD-9) and Common Procedure Terminology, Fourth Edition (CPT-4) codes, were included (Table 1). Cases were defined as individuals with at least one ICD-9 code (727.61, 840.3, 840.4, 840.5, 840.6) or CPT4 code (23410, 23412, 23420, 29827). The definitions of each injury code are listed in Table 1. Phenotypes were defined as: full rupture (individuals with ICD727_61), partial/full rupture (individuals with ICD727_61, CPT23410, CPT23412 or CPT29827), and rotator cuff injury (any code). There were 904 cases of full rupture, 2241 cases of partial/full rupture and 8357 cases of rotator cuff injury from the cohort of 102,979 individuals. Participants were categorized as cases if they contained any of the injury codes listed in Table 1; otherwise, they were categorized as controls.

**Table 1:** Rotator cuff injury phenotypes classified by ICD and/or CPT codes used in genome-wide association analyses.

Description	Code^a^	N (%)^b^
Complete rupture of rotator cuff	ICD727_61	904 (7.4)
Infraspinatus sprain	ICD840_3	73 (0.6)
Rotator cuff sprain	ICD840_4	7808 (63.6)
Subscapularis sprain	ICD840_5	210 (1.7)
Supraspinatus sprain	ICD840_6	709 (5.8)
Repair of ruptured rotator cuff (acute)	CPT23410	324 (2.6)
Repair of ruptured rotator cuff (chronic)	CPT23412	420 (3.4)
Reconstruction of complete rotator cuff avulsion	CPT23420	244 (2.0)
Shoulder arthroscopy with rotator cuff repair	CPT29827	1587 (12.9)
**Total**		12279 (100)

^a^ International Statistical Classification of Diseases and Related Health Problems (ICD-9) and Current Procedural Terminology (CPT-4) codes extracted from KPNC electronic health records (EHR) of GERA cohort subjects.

^b^ Number of instances of each specific code in the EHR, with percent of the total number of codes in parentheses. The total number of codes (n = 12279) exceeds the total number of cases (n = 8357) since many individuals (n = 2395) have multiple codes in their health records.

### Genome-wide association and meta-analysis

Genome-wide association analyses of the GERA cohort genomic data were conducted using PLINK v1.90(b3.34) (https://www.cog-genomics.org/plink2)[16,17]. SNP associations were tested with rotator cuff injury codes with a logistic regression model using allele counts for typed and imputed SNPs in an additive genetic model for each of the five race/ethnic populations. The model was adjusted for genetic sex, age at enrollment into the RPGEH cohort, race/ethnicity using principal components, and variations in genotyping protocol. The final number of SNPs that were analyzed was 9,870,147 for European (EUR); 11,158,335 for Latin American (LAT); 8,951,026 for East Asian (EAS); 17,224,907 for African American (AFR); and 25,874 for Southeast Asian (SAS) populations. Results from each population were combined by inverse-variance, fixed-effects meta-analysis. The final number of SNPs that was analyzed in the fixed-effects meta-analysis was 10,582,947.

We examined the level of heterogeneity using two measures: 1) the I^2^ statistic, which measures the percentage of variability across studies that is due to heterogeneity, where a lower value indicates more consistent results across studies, and 2) Cochran’s Q statistic, which measures whether observed differences in study results are due to chance alone, where a low associated p-value indicates heterogeneity[18,19]. Locus plots showing regional association signals were generated in LocusZoom (http://locuszoom.sph.umich.edu/locuszoom)[20].

### Replication analysis of previously reported SNPs

A literature search was conducted to compile a list of candidate genes previously tested for association with rotator cuff injuries. We searched the public databases PubMed/MEDLINE (http://www.ncbi.nlm.nih.gov/pubmed) and Scopus (http://www.scopus.com/) for previously published studies on the genetics of rotator cuff injury. Inclusion criteria for filtering results were: research papers with original data, English language, full-text article available (or abstract with enough data), genetic association studies with human subjects, cases with rotator cuff phenotypes, and gene or SNP associations reported. Articles were included regardless of whether or not the associations were significant and regardless of the size of the study population. Furthermore, the references from each publication were used to find other articles not captured in the initial search. The final searches were conducted on June 1, 2016. Overall, six total publications were identified reporting significant associations for 18 SNPs in 10 genes. Additional information regarding the literature search can be found in the Supplemental Methods. In the replication analysis, we searched our GWAS and meta-analysis results for these SNPs and used the Benjamini-Hochberg method to set the false discovery rate to 5% to account for multiple testing [21].

Summary statistics for all SNPs from the fixed-effects meta-analysis are available at NIH GRASP: https://grasp.nhlbi.nih.gov/FullResults.aspx.

### Ethical considerations

This study analyzed stored data from RPGEH subjects who consented to genomic testing and use of their genomic data, as well as health data from the KPNC Electronic Health Record, for future research studies. The health and genotype data for the subjects were de-identified. All study procedures were approved by the Kaiser Permanente Research Institute Institutional Review Board.

## Results

### Study population and genotype information

The Genes, Environment and Health cohort includes genotype and medical data from 102,979 individuals in the Kaiser-Permanente Northern California system. Demographic data for the cohort are presented in Table 2. The electronic health records were interrogated for individuals that had incurred a rotator cuff injury (Table 1)(Methods).

**Table 2:** Demographic factors of the GERA study population used in genome-wide association analyses of rotator cuff injury.

GERA—ALL	Cases^a^	Controls	Overall
**Subjects** [N (%)]	8357 (8.1%)	94622 (91.9%)	102979
**Sex**^**b**^ [N (%)]			
Female	4166 (7.0%)	55505 (93.0%)	59671
Male	4188 (9.7%)	39054 (90.3%)	43242
Undetermined	3 (4.5%)	63 (95.5%)	66
**Race**^**c**^ [N (%)]			
European	6993 (8.4%)	76271 (91.6%)	83264
Latin American	669 (7.8%)	7891 (92.2%)	8560
East Asian	394 (5.2%)	7124 (94.8%)	7518
African American	269 (8.5%)	2892 (91.5%)	3161
South Asian	32 (6.7%)	444 (93.3%)	476
**Age**^**d**^	66.9	62.4	62.7
	(66.7–67.0)	(62.3–62.5)	(62.6–62.8)

^a^ Cases with rotator cuff injury as defined by individuals with 1 or more qualifying ICD-9 or CPT-4 code in their electronic health records (EHR). For details see Methods and Table 2.

^b^ Sex/gender as determined by an individuals genetic data, reported as the number and percentage of total cases, controls, or overall for each respective group. For details see Methods and dbGaP (Study Accession: phs000674.v1.p1).

^c^ Race/ethnic groups as determined by principle component analysis (PCA) on individuals genetic data from the GERA cohort. Reported as the number and percentage of total cases, controls, or overall for each respective group. For details see Methods and dbGaP (Study Accession: phs000674.v1.p1).

^d^ Age at subject enrollment in the GERA cohort, reported as mean age with 95% confidence interval.

### Genome-wide study for association with rotator cuff injury

The RPGEH cohort, genotyping data, methodological approach and logical flow presented here overlap those used in previous work by the same authors on MCL injury, shoulder dislocation, plantar fasciitis, ACL injury, Achilles tendon injury and ankle injury [22–26]. However, the analyses presented here present new results on the genetic basis for rotator cuff injury.

**Fig 1:**
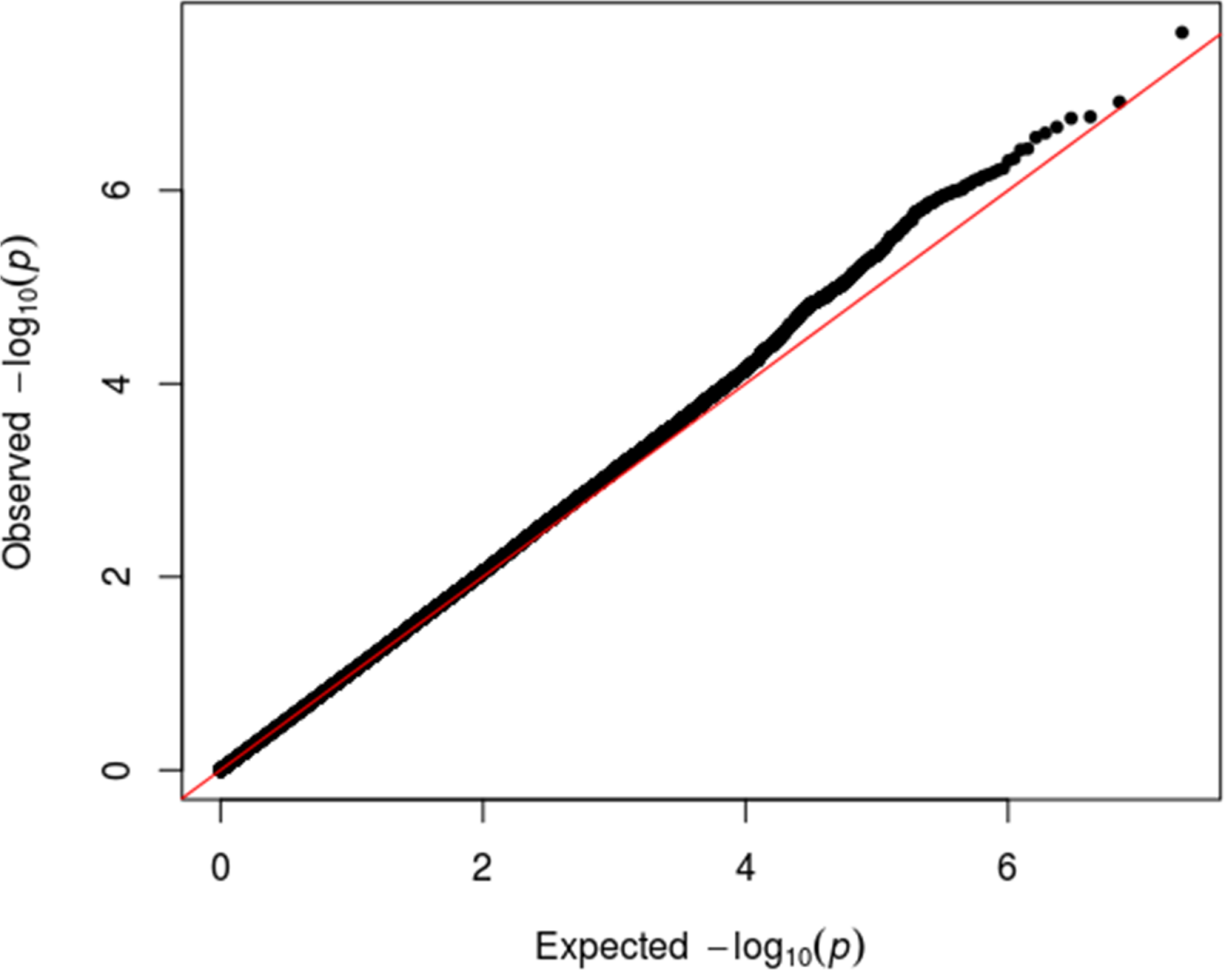
Quantile-quantile plot for genome-wide association analyses of rotator cuff injury. The expected versus observed log transformed values for the 10,582,947 p-values are graphed. The observed p-values (black dots) are plotted on the y-axis and the p-values expected by chance (red line) are plotted on the x-axis.

A logistic regression was performed to search for SNPs associated with rotator cuff injury (Methods). We compared the observed p-values from the GERA cohort meta-analysis to the distribution of p-values that would be expected by chance in a Q-Q plot (Fig 1). We observed slight deviation from the null hypothesis for the lowest observed p-value, illustrated by the farthest upper-right points tending to be above the diagonal red line. This deviation indicates that one or more SNPs from our analysis show an association with rotator cuff injury.

**Fig 2:**
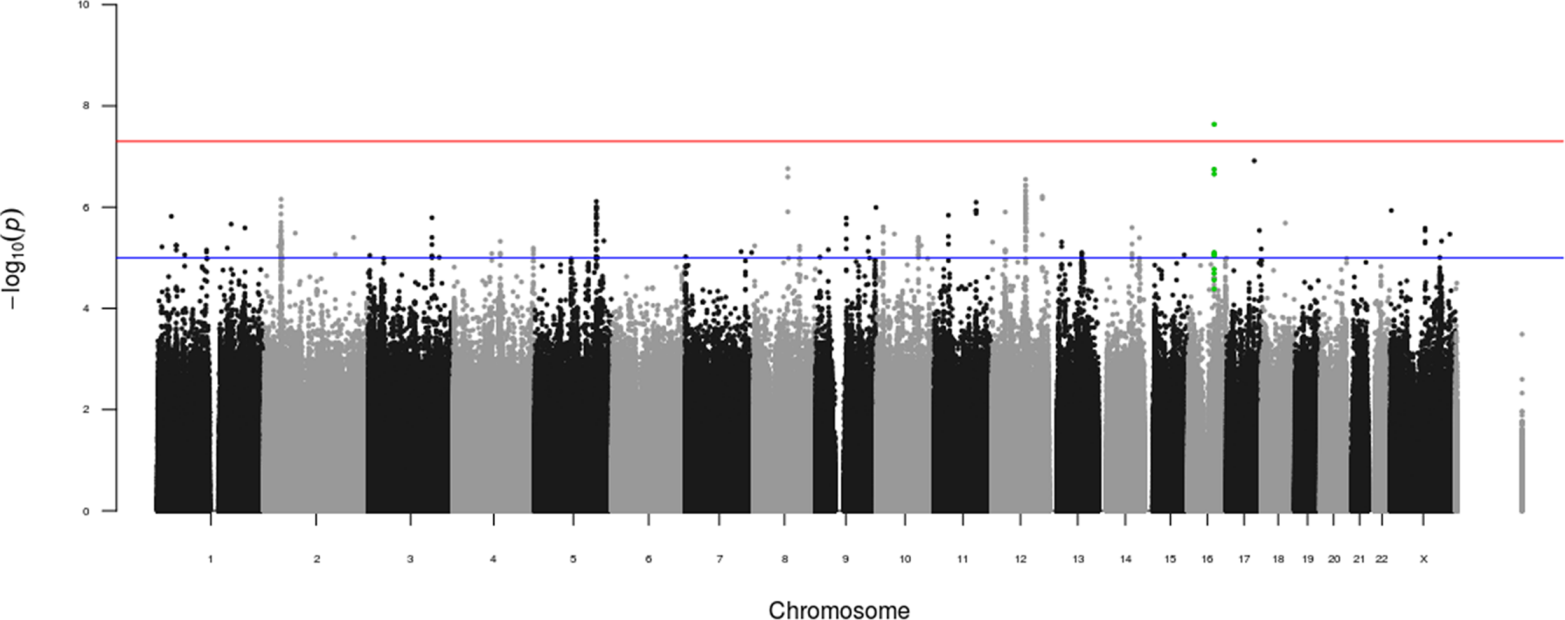
Manhattan plot for genome-wide association analyses of rotator cuff injury. The log_10_ p-values for association with rotator cuff injury for 10,582,947 SNPs from the meta-analysis from this study are plotted by genomic position with chromosome number listed across the bottom. The y-axis shows the -log_10_ p-value for association with rotator cuff injury. The blue line represents suggestive genome-wide significance (p<1x10^-5^) and the red line represents genome-wide significance (p<5x10^-8^). The top SNP rs71404070 and the nine other SNPs within the same linkage disequilibrium block at suggestive significance are highlighted in green.

We plotted the p-value for every SNP from the meta-analysis on a Manhattan plot (Fig 2). The tested SNPs are arranged linearly by genomic position along the x-axis and the p-value for each SNP is indicated along the y-axis. To correct for multiple hypothesis testing, we used the commonly-accepted genome-wide threshold for statistical significance of p<5x10^-8^ (indicated by the red line)[27–29]. One SNP (rs71404070) exceeds the genome-wide statistical threshold for association with rotator cuff injury (Table 3). rs71404070 yielded data for three races (EUR, LAT, AFR) but not the two Asian races. rs71404070 was not directly genotyped in the dataset, but rather the genotypes were imputed with fairly high accuracy (R^2^ = 0.93)(Table 3). Summary statistics for all SNPs from the fixed-effects meta-analysis are available at NIH GRASP: https://grasp.nhlbi.nih.gov/FullResults.aspx.

**Table 3:** Association of rs71404070 with rotator cuff injury from genome-wide association analyses.

SNP	Gene	Allele^a^	P-Value^b^	OR (95% CI)^c^
rs71404070^d^	CDH8/LOC729159	A	2.31x10^-8^	1.25 (1.18–1.33)

^a^ Effect allele.

^b^ P-value from fixed-effects meta-analysis.

^c^ Allelic odds ratio with 95% confidence interval, adjusted for covariates in the logistic regression from fixed-effects meta-analysis.

^d^ rs71404070 was not directly genotyped, but rather the data were imputed with R^2^ = 0.93 (all races), 0.94 (EUR), 0.86 (AFR) and 0.90 (LAT).

**Fig 3:**
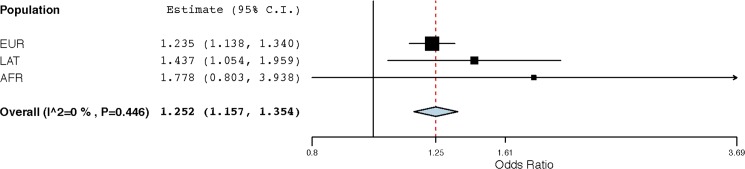
Forest plot for association of rs71404070 with rotator cuff injury. Only three race/ethnic populations yielded data for the lead SNP rs71404070: European, Latin-American and African-American. Effect size estimates with their 95% confidence intervals are given for each individual group, as well as the overall summary result. We observe little heterogeneity as measured by I^2^ (0%) and the p-value for Cochran’s Q statistic (0.446).

The GWAS results were analyzed to determine whether the association with rotator cuff injury for rs71404070 was stronger in some races than in others, a phenomenon known as heterogeneity[30]. A forest plot shows the odds ratio and 95% confidence interval for the three different races with data, as well as the overall result from all three races combined (Fig 3). The odds ratios for each race were in the same direction and of similar magnitude. Using I^2^ and Cochran’s Q to assess heterogeneity, we saw no evidence of heterogeneity; specifically, I^2^ = 0% (95% confidence interval: 0–79%) and Cochran’s Q = 1.614 with p = 0.446.

The allelic odds ratio for rs71404070 is 1.25. Individuals that carried one risk allele at rs71404070 (A/T) had a 29% increased chance of rotator cuff injury compared to individuals that have no risk alleles (Table 4).

**Table 4:** Genotype distributions for rs71404070.

rs71404070	A/A	A/T	T/T
Cases	13	709	7,437
Controls	142	6,213	86,089
Overall	155	6,922	93,526
Risk for rotator cuff injury	8.4%	10.2%	7.9%
Relative risk for rotator cuff injury^a^	1.06	1.29	1.00
	(0.60–1.86)	(1.19–1.40)	

^**a**^ Risk relative to homozygous T/T (95% confidence interval).

Our cohort of rotator cuff injuries includes 904 cases of full rupture and 2241 cases of either full or partial rupture of the rotator cuff (Table 5). We asked whether increasing severity of rotator cuff injury would also increase the strength of the association of rs71404070 with that injury. We repeated the analysis for rs71404070 using either full or partial/full rupture as cases versus uninjured patients as controls (Table 5). We note that the odds ratio increases for the partial/full ruptures (1.38) but not for full ruptures (1.22) compared to the overall cohort of all injured patients (1.25). As expected, the p-values for the association of rs71404070 with each rotator cuff injury phenotype became less significant as the number of cases decreased.

**Table 5:** Association of rs71404070 with full and partial/full rotator cuff tears.

SNP	Phenotype	N^a^	P-Value^b^	OR (95% CI)^c^
rs71404070	All Injuries	8357	2.31x10^-8^	1.25 (1.18–1.33)
rs71404070	Partial or Full Rupture	2241	6.14x10^-5^	1.38 (1.24–1.52)
rs71404070	Full Rupture	904	0.36	1.22 (0.98–1.46)

^a^ Number of cases included in each analysis.

^b^ P-value from fixed-effects meta-analysis.

^c^ Allelic odds ratio with 95% confidence interval, adjusted for covariates in the logistic regression from fixed-effects meta-analysis.

**Fig 4:**
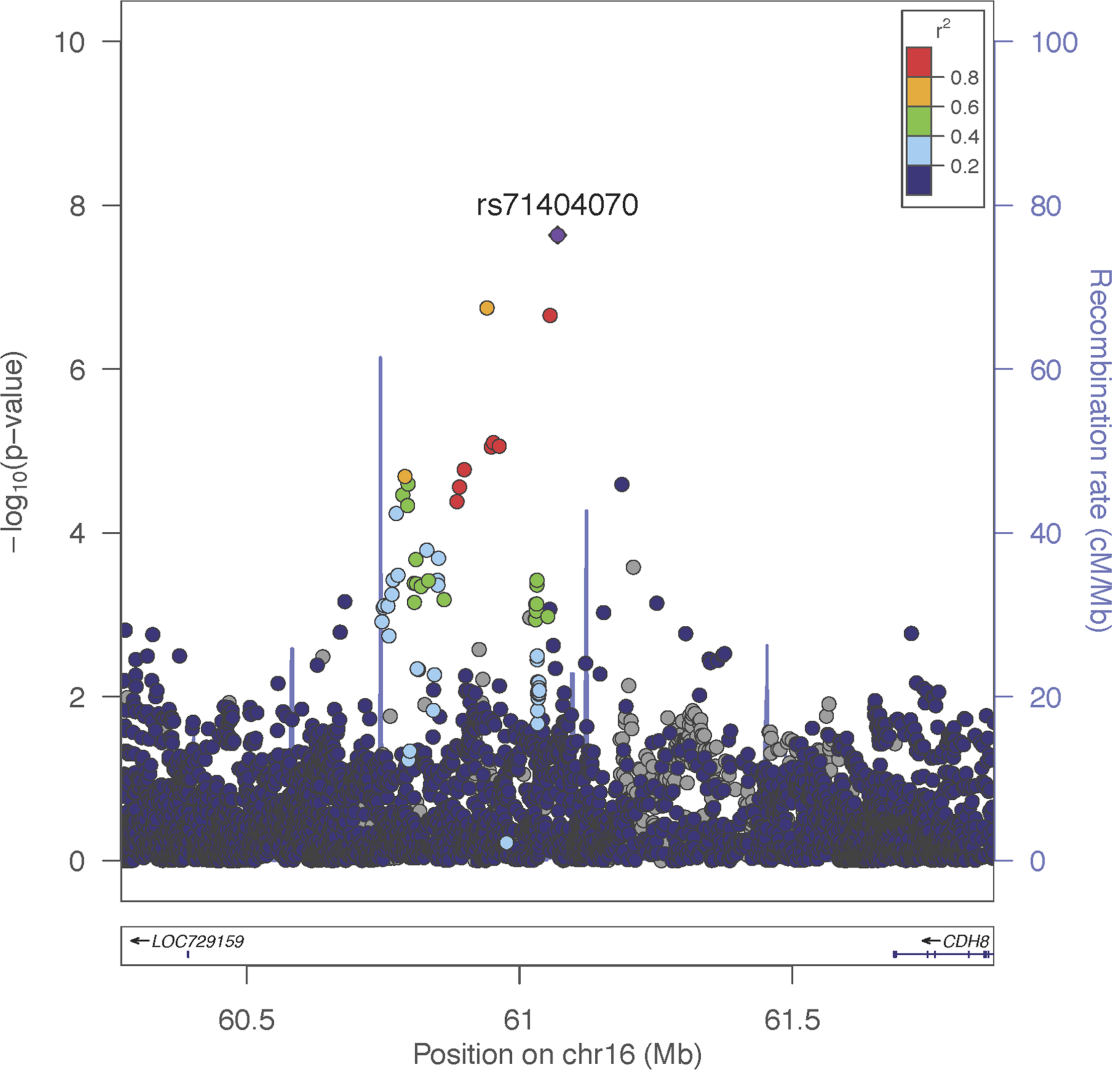
Regional-association plot for rs71404070 with rotator cuff injury. Tested SNPs are arranged by genomic position on chromosome 16 (x-axis) in a 2Mb window around the lead SNP rs71404070, including the locus where our association signal is present. The y-axis indicates -log_10_ p-values for association with rotator cuff injury for each SNP. The top group of SNPs are located in the intergenic region between *LOC729159* and *cadherin8* (*CDH8*). The purple diamond represents the lead SNP rs71404070. The color of dots representing flanking SNPs indicates their linkage disequilibrium (R^2^) with the lead SNP as indicated in the heat map color key. The grey dots represent SNPs that lack R^2^ information in 1000genomes or HapMap. Blue vertical lines show the recombination rate at specific genomic positions and approximate locus boundaries.

Rs71404070 is located in the 1.5 Mb intergenic region between *LOC729159* and *cadherin8* (*CDH8*) on chromosome 16 (Fig 4). There is one SNP that is tightly linked (R^2^ = 0.99) and 8 additional SNPs that are weakly linked (R^2^>0.6) to the sentinel SNP rs71404070 (S1 Table). Since all of these SNPs are linked and tend to be inherited as one haplotype, it is unclear which of these, if any, is the SNP that directly affects risk for rotator cuff injury and which show an association simply because they are linked.

We investigated all of the SNPs linked to rs71404070 for evidence that they may affect the expression or coding capacity of neighboring genes, consistent with a variant that is directly responsible for affecting rotator cuff injury risk. The entire linkage disequilibrium block is intergenic, so none of the SNPs directly affect protein coding regions.

Next, we searched for evidence that these SNPs affect gene expression levels of either *LOC729159* or *cadherin8* by acting as expression quantitative trait loci (eQTLs)(Methods). The Genotype-Tissue Expression database has documented SNPs associated with changes in expression of nearby genes [31]. However, neither rs71404070 nor any of the nine linked SNPs showed an association with variation in expression of either *LOC729159* or *cadherin8*.

Finally, we interrogated data from the ENCODE project to determine whether rs71404070 or any of the nine linked SNPs might affect binding of transcription factors [32]. In general, when transcription factors bind to DNA, they are associated with a factor known as CTCF and the bound region becomes DNAse I hypersensitive[32]. SNPs located in these regions may affect transcription factor binding and consequently gene expression. The lead SNP in our study, rs71404070, was not associated with either CTCF binding or DNAse I hypersensitivity. One weakly-linked SNP, rs35448966 (R^2^ = 0.67), is located in a CTCF binding site[32]. A moderately-linked SNP, rs76226460 (R^2^ = 0.81), is located in a DNase I hypersensitivity peak[32]. These data suggest that these two SNPs might have an effect on expression of nearby genes. However, their association with rotator cuff injury is weak (p>10^−7^) and they are not highly correlated with the sentinel SNP rs71404070 (R^2^<0.81), indicating that these SNPs are not likely to be responsible for the highly-significant association of rs71404070 with rotator cuff injury (S1 Table). In summary, none of the nine SNPs linked to rs71404070 has strong evidence for being directly responsible for affecting the activity of any of the nearby genes. Since rs71404070 has by far the most significant association with rotator cuff injury, it has the best evidence for being the SNP that is responsible for the association.

We attempted to validate the association of rs71404070 with rotator cuff injury using data from a previous GWA study[12]. However, neither rs71404070 itself nor any closely linked SNPs were included in the previous set of genotyped markers (C. Teerlink and R. Tashjian, Personal Communication).

### Failure to validate previously reported SNPs associated with rotator cuff injury

In previous studies, 18 SNPs in 10 genes have been reported to be significantly associated with rotator cuff injury[9–14]. Two SNPs (rs820218 and rs10484958) were identified in a genome-wide screen (with p<1.9 x 10^−7^)[12]. The remaining 16 SNPs were identified in candidate gene studies (with p<0.05)[9–11,13,14]. None of the reported SNPs have been independently replicated for association with rotator cuff injury.

We tested whether any of these 18 SNPs showed an association with rotator cuff injury in our dataset (Table 6). To compensate for multiple testing, we used the Benjamini-Hochberg method, with a false discovery rate of q = 0.05[33,34]. None of the 18 SNPs showed a significant association with rotator cuff injury in our dataset. In previous studies, the cases had full ruptures of the rotator cuff whereas in this work, the cases included partial ruptures, full ruptures, sprains and avulsions. One possibility is that the 18 SNPs might show an association with full ruptures but not with other injuries of the rotator cuff. To address this possibility, we repeated the analysis using only full ruptures (904 cases) or partial/full ruptures (2241 cases) of the rotator cuff (Table 6). Even restricting the analysis to full and/or partial ruptures, none of the 18 previous SNPs showed a significant association with rotator cuff injury in our dataset.

**Table 6:** Replication testing of SNPs previously reported to be associated with rotator cuff injury.

			All RTC^a^		Partial/Full Rupture^b^		Full Rupture^c^		
SNP	Gene	EA^d^	P^e^	OR	P^e^	OR	P^e^	OR	Ref^g^
				(95% CI^)^^f^		(95% CI)^f^		(95% CI)^f^	
rs3045	ANKH	C	0.78	1.01	0.71	1.02	0.72	0.97	[11]
				(0.95–1.07)		(0.92–1.12)		(0.82–1.12)	
rs1800972	DEFB1	C	0.53	0.99	0.08	0.93	0.05	0.89	[10]
				(0.95–1.03)		(0.85–1.01)		(0.78–0.99)	
rs17583842	ESRRB	C	0.014	0.95	0.88	1.01	0.16	1.08	[12,13]
				(0.91–0.99)		(0.94–1.08)		(0.97–1.19)	
rs4903399	ESRRB	T	0.86	1.00	0.87	0.99	0.66	1.03	[10]
				(0.96–1.04)		(0.92–1.06)		(0.90–1.16)	
rs1676303	ESRRB	C	0.16	0.96	0.02	0.89	0.43	0.94	[10]
				(0.91–1.01)		(0.79–0.99)		(0.80–1.08)	
rs12574452	FGF3	A	0.95	1.00	0.14	1.06	0.64	1.03	[10]
				(0.96–1.04)		(0.99–1.13)		(0.90–1.16)	
rs1011814	FGF10	T	0.8	1.00	0.19	0.96	0.43	0.96	[10]
				(0.97–1.03)		(.90–1.02)		(0.86–1.06)	
rs11750845	FGF10	T	0.7	0.99	ND^h^	ND	ND	ND	[10]
				(0.96–1.02)					
rs13317	FGFR1	C	0.75	1.01	0.11	0.94	0.18	0.92	[10]
				(0.97–1.05)		(0.86–1.02)		(0.79–1.02)	
rs820218	SAP30BP	A	0.24	0.98	0.22	0.96	0.85	0.99	[12]
				(0.94–1.02)		(0.89–1.03)		(0.89–1.11)	
rs10484958	SASH1	A	0.85	1.00	0.03	0.91	0.10	0.89	[12]
				(0.95–1.05)		(0.82–1.00)		(0.77–1.03)	
rs4654760	TNAP	T	0.83	1.01	0.33	0.93	0.07	1.21	[11]
				(0.94–1.08)		(0.79–1.07)		(0.98–1.50)	
rs1138545	TNC	T	0.73	1.01	0.99	1.00	0.72	1.03	[14]
				(0.96–1.06)		(0.91–1.09)		(0.89–1.16)	
rs3789870	TNC	A	0.93	1.00	0.87	0.99	ND	ND	[14]
				(0.96–1.04)		(0.92–1.06)			
rs10759753	TNC	G	0.91	1.00	0.93	1.00	0.70	0.98	[14]
				(0.96–1.045)		(0.93–1.07)		(0.88–1.09)	
rs72758637	TNC	G	0.62	1.01	0.95	1.00	0.81	1.02	[14]
				(0.96–1.06)		(0.91–1.09)		(0.88–1.117)	
rs7021589	TNC	C	0.64	1.01	0.86	1.01	ND	ND	[14]
				(0.95–1.06)		(0.92–1.10)			
rs7035322	TNC	A	0.72	1.01	0.93	1.00	ND	ND	[14]
				(0.97–1.05)		(0.93–1.07)			

^a^ All Rotator Cuff Injuries; 8357 cases.

^b^ Partial or Full Rotator Cuff Tears; 2241 cases.

^c^ Full Rotator Cuff Tears; 904 cases.

^d^ Effect allele from this study, also denoted A1 in GWAS + meta-analysis results.

^e^ P-value from fixed-effects meta-analysis in this study.

^f^ Allelic odds ratio with 95% confidence interval, adjusted for covariates in the logistic regression, from fixed-effects meta-analysis in this study.

^g^ Original publication showing an association of the candidate SNP with rotator cuff injury.

^h^ No Data for this SNP.

## Discussion

With the advent of large-scale genotyping programs, it is now possible to screen the entire genome for polymorphisms associated with musculoskeletal injury risks, such as rotator cuff tears. A genome-wide screen for rotator cuff injury could provide new insights regarding the differences between individuals in their inherent propensity for injury and reveal insights into underlying mechanisms for shoulder tendinopathy. Furthermore, because the genotype data includes most of the common polymorphisms that are known, a genome-wide screen reports the strongest associations in the genome in an unbiased manner. Here, we have performed a study to find DNA polymorphisms associated with rotator cuff injury by obtaining access to large-scale genotype and phenotype data from the Research Program on Genes, Environment and Health. The data contained information from 102,979 individuals of whom 8,357 had rotator cuff injury.

We found rs71404070 to be associated with rotator cuff injury with a p-value that has genome-wide significance. It will be important to validate this finding in independent studies[35,36]. rs71404070 lies in a linkage disequilibrium block that contains nine other SNPs that also show an association with rotator cuff injury, although at a lower level than rs71404070. Within this block, it is unclear which variant(s) causes increased risk of rotator cuff injury and which are neutral polymorphisms linked to this variant(s). Neither rs71404070 nor any of the linked SNPs affect protein-coding regions. Additionally, none of these SNPs are known to affect expression of the two nearest genes (*LOC729159* or *cadherin8*). The *cadherin8* gene encodes a type II classical cadherin, which is an integral membrane protein that mediates calcium-dependent cell-cell adhesion. The function of LOC729159 is currently unknown.

Individuals carrying one risk allele at rs71404070 (A/T) had a 29% increased chance of injury compared to individuals with no risk allele (T/T). The size of the effect of rs71404070 is typical for many genome-wide association studies but far smaller than those for simple Mendelian traits. The rs71404070 genotype explains part of the heritable risk for rotator cuff injury, but a large part of the heritability remains unanswered. For some traits such as height or bone mineral density, heritability is largely explained by the cumulative effect from hundreds or thousands of loci [37–39]. One possibility is that rotator cuff injury is also polygenic, in which case identification of more loci in future studies might explain the heritability of this injury more fully. Another possibility is that rotator cuff injury may be heterogeneous; i.e. there may be distinct types of injury, and the rs71404070 SNP might explain risk for some types but not others [40]. In this case, methods could be developed to classify rotator cuff injury into sub-types, in which case the effect size of rs71404070 might increase for a specific sub-type of injury.

We re-tested 18 SNPs in 10 genes that were previously reported to show an association with rotator cuff injury, but we were unable to validate any associations. Evidence from many other genetic association studies suggests that candidate gene associations need to be independently replicated, otherwise their credibility is low[41–44]. Our study included 8,357 cases of rotator cuff injury compared to between 175 and 331 cases of rotator cuff injury in previous studies[12,13]. Compared to previous studies, our study had a larger number of cases, indicating that we had good statistical power to replicate the previously reported associations[45]. For example, for a SNP with a minor allele frequency of 5% and a genotype relative risk of 1.2 for 8,357 cases, power calculations indicate that our study had a 99% chance of replication. However, if we restrict the re-testing to just 904 cases of full rotator cuff rupture, our study had only a 15% chance of replicating such a SNP.

One possible reason explaining why our study was not able to replicate previous results is that rotator cuff injury was classified according to data from electronic health records. Some of the previous studies classified rotator cuff injuries using magnetic resonance imaging to confirm full-thickness rotator cuff tears. For large studies like ours, it is typical to perform bioinformatics searches of electronic health records, as it is not possible to manually evaluate each case. Nevertheless, it could be that some of the rotator cuff injuries in our study may be misclassified due to inaccuracy of the electronic health records.

The summary statistics for 10,582,947 SNPs from the GWAS for rotator cuff injury may be used in future genetic studies of rotator cuff injury. For instance, one could make a list of additional genes and genetic pathways involved in the development, formation or structure of the rotator cuff, and then use the data presented here to test those genes for an association. In addition, GWA studies for rotator cuff injury may be conducted in the future, in which case the data from this paper could be used to validate any new SNPs that are found. Finally, the data on rotator cuff injury could be combined with data from other musculoskeletal injuries (e.g. Achilles tendon injury) in a cross-phenotype meta-analysis in order to find SNPs associated with tendon injuries in general.

An attractive possibility is that rs71404070 could be used as a diagnostic marker to identify individuals with increased risk for injury, and then to take preventative measures to alleviate some of that risk. In our data from the general population, having one copy of the risk A allele of rs71404070 increased the risk for rotator cuff injury by 29% compared to having no copies. The underlying biological mechanism responsible for the association of rs71404070 with rotator cuff injury is currently unknown.

There are several limitations to this study. First, the results should be replicated in an independent cohort. Second, the phenotypes were defined from codes contained in patient electronic health records, which may be inaccurate. Third, the number of individuals of Latin-American, African-American and Asian ethnicity was relatively small, and hence the association results for these results are weaker than those from the European group. Fourth, these results should be repeated in a cohort of athletes to determine whether the effect size is similar in athletes. Fifth, additional studies are warranted to begin to illuminate the underlying biological mechanism for the association of variation near *cadherin8* and rotator cuff injury.

## Supporting information

S1 Table Data for SNPs linked to sentinel SNP rs71404070. (DOCX)

S1 Methods Supplemental methods. (DOCX)

## References

[1] Yamamoto A, Takagishi K, Osawa T, Yanagawa T, Nakajima D, Shitara H, et al (2010) Prevalence and risk factors of a rotator cuff tear in the general population. J Shoulder Elbow Surg 19: 116–120. doi: 10.1016/j.jse.2009.04.006 19540777 10.1016/j.jse.2009.04.006

[2] Tashjian RZ (2012) Epidemiology, natural history, and indications for treatment of rotator cuff tears. Clin Sports Med 31: 589–604. doi: 10.1016/j.csm.2012.07.001 23040548 10.1016/j.csm.2012.07.001

[3] Connor PM, Banks DM, Tyson AB, Coumas JS, D'Alessandro DF (2003) Magnetic resonance imaging of the asymptomatic shoulder of overhead athletes: a 5-year follow-up study. Amer J Sports Med 31: 724–727. 12975193 10.1177/03635465030310051501

[4] Lewis JS (2010) Rotator cuff tendinopathy: a model for the continuum of pathology and related management. Br J Sports Med 44: 918–923. doi: 10.1136/bjsm.2008.054817 19364757 10.1136/bjsm.2008.054817

[5] Economopoulos KJ, Brockmeier SF (2012) Rotator cuff tears in overhead athletes. Clin Sports Med 31: 675–692. doi: 10.1016/j.csm.2012.07.005 23040553 10.1016/j.csm.2012.07.005

[6] Williams GR, Kelley M (2000) Management of rotator cuff and impingement injuries in the athlete. J Athl Train 35: 300–315. 16558644 PMC1323393

[7] Gwilym SE, Watkins B, Cooper CD, Harvie P, Auplish S, Pollard TCB, et al (2009) Genetic influences in the progression of tears of the rotator cuff. J Bone Joint Surg Br 91: 915–917. doi: 10.1302/0301-620X.91B7.22353 19567856 10.1302/0301-620X.91B7.22353

[8] Harvie P, Ostlere SJ, Teh J, McNally EG, Clipsham K, Burston BJ, et al (2004) Genetic influences in the aetiology of tears of the rotator cuff. Sibling risk of a full-thickness tear. J Bone Joint Surg Br 86: 696–700. 15274266 10.1302/0301-620x.86b5.14747

[9] Bonato LL, Quinelato V, Pinheiro AdR, Amaral MVG, de Souza FN, Lobo JC, et al (2016) ESRRB polymorphisms are associated with comorbidity of temporomandibular disorders and rotator cuff disease. Int J Oral Maxillofac Surg 45: 323–331. doi: 10.1016/j.ijom.2015.10.007 26584852 10.1016/j.ijom.2015.10.007

[10] Motta GR, Amaral MV, Rezende E, J. (2014) Evidence of genetic variations associated with rotator cuff disease. Elbow Surg 23: 227–235. 10.1016/j.jse.2013.07.053 24129055

[11] Peach CA, Zhang Y, Dunford JE, Brown MA, Carr AJ (2007) Cuff tear arthropathy: evidence of functional variation in pyrophosphate metabolism genes. Clin Orthop Relat Res 462: 67–72. doi: 10.1097/BLO.0b013e31811f39de 17563703 10.1097/BLO.0b013e31811f39de

[12] Tashjian RZ, Granger EK, Farnham JM, Cannon-Albright LA, Teerlink CC (2016) Genome-wide association study for rotator cuff tears identifies two significant single-nucleotide polymorphisms. J Shoulder Elbow Surg 25: 174–179. doi: 10.1016/j.jse.2015.07.005 26350878 10.1016/j.jse.2015.07.005

[13] Teerlink CC, Cannon-Albright LA, Tashjian RZ (2015) Significant association of full-thickness rotator cuff tears and estrogen-related receptor-β (ESRRB). J Shoulder Elbow Surg 24: e31–35. doi: 10.1016/j.jse.2014.06.052 25219474 10.1016/j.jse.2014.06.052

[14] Kluger R, Burgstaller J, Vogl C, Brem G, Skultety M, Mueller S (2016) A candidate gene approach identifies six SNPs in tenascin-C (TNC) associated with degenerative rotator cuff tears. J Orthop Res. 10.1002/jor.23321 27248364

[15] Jorgenson E, Makki N, Shen L, Chen DC, Tian C, Eckalbar WL, et al (2015) A genome-wide association study identifies four novel susceptibility loci underlying inguinal hernia. Nat Commun 6: 10130 doi: 10.1038/ncomms10130 26686553 10.1038/ncomms10130 PMC4703831

[16] Chang CC, Chow CC, Tellier LC, Vattikuti S, Purcell SM, Lee JJ (2015) Second-generation PLINK: rising to the challenge of larger and richer datasets. Gigascience 4: 7 doi: 10.1186/s13742-015-0047-8 25722852 10.1186/s13742-015-0047-8 PMC4342193

[17] Purcell S, Neale B, Todd-Brown K, Thomas L, Ferreira MAR, Bender D, et al (2007) PLINK: a tool set for whole-genome association and population-based linkage analyses. Am J Hum Genet 81: 559–575. doi: 10.1086/519795 17701901 10.1086/519795 PMC1950838

[18] Cochran WG (1954) The combination of estimates from different experiments. Biometrics 10: 101–129.

[19] Higgins JPT, Thompson SG, Deeks JJ, Altman DG (2003) Measuring inconsistency in meta-analyses. Br Med J (Clin Res Ed) 327: 557–560. 10.1136/bmj.327.7414.557 PMC192859 12958120

[20] Pruim RJ, Welch RP, Sanna S, Teslovich TM, Chines PS, Gliedt TP, et al (2010) LocusZoom: regional visualization of genome-wide association scan results. Bioinformatics (Oxford, England) 26: 2336–2337. 10.1093/bioinformatics/btq419 PMC2935401 20634204

[21] Benjamini Y, Drai D, Elmer G, Kafkafi N, Golani I (2001) Controlling the false discovery rate in behavior genetics research. Behav Brain Res 125: 279–284. 11682119 10.1016/s0166-4328(01)00297-2

[22] Kim SK, Avins AL, Kleimeyer JP, Fredericson M, Ioannidis JP, Dragoo JL, et al (2017) Genome-wide Association Study Reveals Two Genetic Variants Associated with Plantar Fasciitis or Fibromatosis. Int J Sports Med in press. 10.1055/s-0044-100280 29534260

[23] Kim SK, Kleimeyer JP, Ahmed MA, Avins AL, Fredericson M, Dragoo JL, et al (2017) A Genetic Marker Associated with Shoulder Dislocation. Int J Sports Med 38: 508–514. doi: 10.1055/s-0043-106190 28521375 10.1055/s-0043-106190

[24] Kim SK, Roos TR, Roos AK, Kleimeyer JP, Ahmed MA, Goodlin GT, et al (2016) Genome-wide Association Screens for Achilles Tendon and ACL Tears and Tendinopathy PLoS One 12: e0170422. 10.1371/journal.pone.0170422 PMC5373512 28358823

[25] Roos AK, Avins AL, Ahmed MA, Kleimeyer JP, Roos TR, Fredericson M, et al (2017) Two Genetic Loci Associated with Medial Collateral Ligament Injury. Int J Sports Med 38: 501–507. doi: 10.1055/s-0043-104853 28482362 10.1055/s-0043-104853

[26] Kim SK, Kleimeyer JP, Ahmed MA, Avins AL, Fredericson M, Dragoo JL, et al (2017) Two Genetic Loci Associated with Ankle Injury. PLoS One submitted. 10.1371/journal.pone.0185355 PMC5619760 28957384

[27] Hoggart CJ, Clark TG, De Iorio M, Whittaker JC, Balding DJ (2008) Genome-wide significance for dense SNP and resequencing data. Genet Epidemiol 32: 179–185. doi: 10.1002/gepi.20292 18200594 10.1002/gepi.20292

[28] Panagiotou OA, Ioannidis JPA, Genome-Wide Significance P (2012) What should the genome-wide significance threshold be? Empirical replication of borderline genetic associations. Int J Epidemiol 41: 273–286. doi: 10.1093/ije/dyr178 22253303 10.1093/ije/dyr178

[29] Pe'er I, Yelensky R, Altshuler D, Daly MJ (2008) Estimation of the multiple testing burden for genomewide association studies of nearly all common variants. Genet Epidemiol 32: 381–385. doi: 10.1002/gepi.20303 18348202 10.1002/gepi.20303

[30] Ioannidis JPA, Patsopoulos NA, Evangelou E (2007) Heterogeneity in meta-analyses of genome-wide association investigations. PloS One 2: e841 doi: 10.1371/journal.pone.0000841 17786212 10.1371/journal.pone.0000841 PMC1950790

[31] Consortium GT (2015) Human genomics. The Genotype-Tissue Expression (GTEx) pilot analysis: multitissue gene regulation in humans. Science 348: 648–660. doi: 10.1126/science.1262110 25954001 10.1126/science.1262110 PMC4547484

[32] Consortium EP (2012) An integrated encyclopedia of DNA elements in the human genome. Nature 489: 57–74. doi: 10.1038/nature11247 22955616 10.1038/nature11247 PMC3439153

[33] Bland JM, Altman DG (1995) Multiple significance tests: the Bonferroni method. BMJ (Clinical research ed) 310: 170. 10.1136/bmj.310.6973.170 PMC2548561 7833759

[34] Storey JD, Tibshirani R (2003) Statistical significance for genomewide studies. Proc Natl Acad Sci U S A 100: 9440–9445. doi: 10.1073/pnas.1530509100 12883005 10.1073/pnas.1530509100 PMC170937

[35] Kraft P, Zeggini E, Ioannidis JPA (2009) Replication in genome-wide association studies. Stat Sci 24: 561–573. doi: 10.1214/09-STS290 20454541 10.1214/09-STS290 PMC2865141

[36] Nhgri(2007) NCI-Group on Replication in Association Studies. Replicating genotypephenotype associations Nature 447: 655–660. doi: 10.1038/447655a17554299

[37] Boyle EA, Li YI, Pritchard JK (2017) An Expanded View of Complex Traits: From Polygenic to Omnigenic. Cell 169: 1177–1186. doi: 10.1016/j.cell.2017.05.038 28622505 10.1016/j.cell.2017.05.038 PMC5536862

[38] Wood AR, Esko T, Yang J, Vedantam S, Pers TH, Gustafsson S, et al (2014) Defining the role of common variation in the genomic and biological architecture of adult human height. Nat Genet 46: 1173–1186. doi: 10.1038/ng.3097 25282103 10.1038/ng.3097 PMC4250049

[39] Kemp JP, Morris JA, Medina-Gomez C, Forgetta V, Warrington NM, Youlten SE, et al (2017) Identification of 153 new loci associated with heel bone mineral density and functional involvement of GPC6 in osteoporosis. Nat Genet. 10.1038/ng.3949 PMC5621629 28869591

[40] Hodge SE, Greenberg DA (2016) How Can We Explain Very Low Odds Ratios in GWAS? I. Polygenic Models. Hum Hered 81: 173–180. doi: 10.1159/000454804 28171865 10.1159/000454804

[41] Ioannidis JP, Ntzani EE, Trikalinos TA, Contopoulos-Ioannidis DG (2001) Replication validity of genetic association studies. Nature genetics 29: 306–309. doi: 10.1038/ng749 11600885 10.1038/ng749

[42] Ioannidis JP, Tarone R, McLaughlin JK (2011) The false-positive to false-negative ratio in epidemiologic studies. Epidemiology 22: 450–456. doi: 10.1097/EDE.0b013e31821b506e 21490505 10.1097/EDE.0b013e31821b506e

[43] Moonesinghe R, Khoury MJ, Liu T, Ioannidis JPA (2008) Required sample size and nonreplicability thresholds for heterogeneous genetic associations. Proceedings of the National Academy of Sciences of the United States of America 105: 617–622. doi: 10.1073/pnas.0705554105 18174335 10.1073/pnas.0705554105 PMC2206585

[44] Siontis KCM, Patsopoulos NA, Ioannidis JPA (2010) Replication of past candidate loci for common diseases and phenotypes in 100 genome-wide association studies. European journal of human genetics: EJHG 18: 832–837. doi: 10.1038/ejhg.2010.26 20234392 10.1038/ejhg.2010.26 PMC2987361

[45] Zeggini E, Ioannidis JPA (2009) Meta-analysis in genome-wide association studies. Pharmacogenomics 10: 191–201. doi: 10.2217/14622416.10.2.191 19207020 10.2217/14622416.10.2.191 PMC2695132

