# Supplementary material for: Genome-wide association study identifies a locus associated with rotator cuff injury
Source: PLoS One. 2017 Dec 11;12(12):e0189317. doi: 10.1371/journal.pone.0189317 (PMC5724859; doi:10.1371/journal.pone.0189317)
Supplement: S1 Table — (DOCX) [file pone.0189317.s001.docx]

**S1 Table. Data for SNPs linked to sentinel SNP rs71404070.**

| BP^a^ | SNP^b^ | P-value^c^ | OR (95% CI)^d^ | R^2 e^ | D' ^f^ |
| --- | --- | --- | --- | --- | --- |
| **61069815** | **rs71404070** | **2.31x10^-08^** | **1.25 (1.18-1.33)** | **1.00** | **1.00** |
| 61055861 | rs71404068 | 2.21x10^-07^ | 1.22 (1.14-1.29) | 0.99 | 1.00 |
| 60962533 | rs34670482 | 8.70x10^-06^ | 1.17 (1.10-1.24) | 0.68 | 0.96 |
| 60952010 | rs12920571 | 7.87x10^-06^ | 1.17(1.10-1.24) | 0.67 | 0.96 |
| 60948036 | rs35701042 | 8.88x10^-06^ | 1.17 (1.10-1.24) | 0.67 | 0.96 |
| 60940189 | rs76226460 | 1.79x10^-07^ | 1.27 (1.18-1.35) | 0.81 | 0.95 |
| 60898547 | rs34706885 | 1.68x10^-05^ | 1.16 (1.09-1.23) | 0.67 | 0.96 |
| 60889902 | rs12928670 | 2.74x10^-05^ | 1.16 (1.09-1.23) | 0.67 | 0.95 |
| 60885151 | rs34140721 | 4.13x10^-05^ | 1.16 (1.09-1.23) | 0.67 | 0.95 |
| 60790088 | rs35448966 | 2.04x10^-05^ | 1.17 (1.10-1.25) | 0.67 | 0.86 |

^a^ Position on chromosome 16 (HG18).

^b^ Tag SNP for the sentinel SNP rs71404070.

^c^ P-value from fixed-effects meta-analysis for association with rotator cuff injury.

^d^ Odds ratio (95% confidence interval).

^e^ R^2^ for linkage to sentinel SNP rs71404070.

^f^ D’ for linkage to sentinel SNP rs71404070.
