# Supplementary material for: Genome-wide association study identifies a locus associated with rotator cuff injury
Source: PLoS One. 2017 Dec 11;12(12):e0189317. doi: 10.1371/journal.pone.0189317 (PMC5724859; doi:10.1371/journal.pone.0189317)
Supplement: S1 Methods — (DOCX) [file pone.0189317.s002.docx]

**Supplemental Methods**

**GERA Cohort**

The Genetic Epidemiology Research on Adult Health and Aging (GERA) cohort is comprised of 110,266 adult members of Kaiser Permanente Northern California (KPNC) Medical Care Plan. It is a component of the KPNC Research Program on Genes, Environment and Health (RPGEH). A complete description of the cohort and study design can be found in dbGaP (Study Accession: phs000674.v1.p1). Participants were enrolled through a mailed survey of about 1.9 million adult members of KPNC in 2007. A total of 435,983 members completed the survey and in 2008 were provided a consent form authorizing use of biospecimens, survey data, and Electronic Health Record (EHR) data for use in studies of genetic and environmental influences on health and disease. Respondents who completed consent forms were mailed saliva collection kits. A total of 109,837 participant samples were selected for genotyping, 103,067 samples passed quality control, and we received full data for 102,979 samples. The average age of the participants at the time of sample collection was 62.9 years old (±13.8 years). Length of membership in KPNC averaged 23.5 years. The medical record contains the entire patient history, including injuries that occurred prior to enrollment in KPNC.

Our analysis cohort (n=102,979) includes 59,671 females, 43,242 males, and 66 individuals of ambiguous sex (Table 1). Moreover, our analysis cohort is ethnically diverse, including 83,264 European-White (EUR); 8,560 Latino (LAT); 7,518 East Asian (EAS); 3,161 African-American (AFR); and 476 South Asian (SAS) individuals based on ancestry principle components. The group includes 3,741 parent-child, 2018 sibling-sibling, and 34 identical twin pairs.

**Genotyping, Quality Control, Relatedness, Imputation, Genetic Ancestry**

Participants were previously genotyped at over 650,000 SNPs on four race/ethnicity specific Affymetrix Axiom genome-wide arrays optimized for individuals of European, African-American, East Asian, and Latino race/ethnicity.^5, 6^ The arrays were designed using human genome b36, but the probesets were remapped to b37. The EUR and EAS arrays were designed for Axiom v1 reagents (kit A) and mostly processed accordingly. The AFR and LAT arrays were designed for Axiom v2 reagents (kit O) and processed accordingly.

Genotype quality control procedures for the GERA cohort were performed on an array-wise basis, as described previously.^11^ Samples were excluded based on bad dish quality control (<0.82) and/or low initial genotype call rate (<0.97). To improve genotype calls, SNPs were re-called within packages of plates assayed under similar conditions (array type, reagent, hibernation time, and DNA concentration). SNPs were removed if either package call rate or overall call rate across packages was low (<90%). Additional SNP exclusion criteria are described in Kvale et al. 2015.^11^ Overall, >94% of samples and >98% SNPs passed quality control. The final number of SNPs that were directly genotyped was 668,265 for EUR; 813,551 for LAT; 709,104 for EAS; and 889,338 for AFR arrays.

Relatedness among participants was determined using King Robust v1.4 (<http://people.virginia.edu/~wc9c/KING/>).^13^ Using the set of 144,799 high-performing SNPs common across all four arrays and standard thresholds (call rate >99.5%), relationships among participants were uniquely characterized as monozygotic twins, siblings, or parent-child.

Genotypes were pre-phased with Shape-IT v2.r644 (https://mathgen.stats.ox.ac.uk/genetics_software/shapeit/shapeit.html)^9^ then imputed to a cosmopolitan reference panel consisting of all individuals from the 1000 Genomes Project (Mar 2012 release)^8^ using IMPUTE2 v2.2.2 (https://mathgen.stats.ox.ac.uk/impute/impute_v2.html)^9^ and standard procedures with a cutoff of R^2^ > 0.3.^7^ The quality of the imputed data was previously validated in Jorgenson et al., 2015 ^10^. The final number of imputed genetic markers accepted for analysis was 31,085,734. Arrays were merged to create a single file and then broken into smaller genomic chunks in the standard IMPUTE2 probabilistic format with a corresponding PLINK family file. The estimated R^2^-info metric from IMPUTE2 estimates the correlation between the true and imputed genotype and was used to filter SNPs in our GWA analyses.^14^

Determination of genetic ancestry was performed by principal component analysis (PCA)^1^, using the smartpca program from EIGENSOFT v4.2 (https://github.com/DReichLab/EIG).^15^ PCA was performed separately for individuals genotyped on the four different arrays. Eigenvectors were created using 41,228 high-quality SNPs common amongst all arrays^12^ and the Human Genome Diversity Project (HGDP) (dbGaP Study Accession: phs000674.v1.p1).^3^ All individuals from the LAT, EAS, AFR arrays were included. For the EUR array, 20,000 individuals were selected for PCA and the results used to project the remaining subjects. These ancestry principal components were used in the GWAS to adjust for genetic ancestry.

**Genome-Wide Association and Meta-Analysis**

In addition to the above quality control and before performing GWAS, SNPs were filtered to exclude those with: 1) low quality imputation (r^2^<0.3); 2) minor allele frequency below 0.5% (MAF<0.005); and 3) substantial deviation from Hardy-Weinberg equilibrium (HWE p<1x10^-50^) using a mid-point p-value correction.^4^ Individuals were excluded if sex was ambiguous (n=66). The variations in genotyping protocol include: chip type for all populations (refers to the Affymetrix chip version), genotyping package (refers to the set of chips that were processed together) and reagent kit (refers to A vs O reagent kit distributed by Affymetrix) when there was variation in the individuals of a specific population. For the EUR population the first 10 ancestry principal components were used as covariates. For the EAS, AFR, LAT, SAS populations, the first 6 ancestry principal components were used. To assess for inflation due to population stratification, the genomic control parameter (λ) was calculated using R v3.2.4 (www.r-project.org).^16^ Genomic inflation was small for GWA analyses in the EUR, LAT, EAS, AFR populations (λ<1.032), but substantial in the SAS population (λ~0.405). Subsequently, p-values were adjusted for genomic control in each population. SNPs found in only one population were included in the analysis, but SNPs with minor allele mismatches between populations were excluded.

We report all the SNP associations from the fixed-effects meta-analysis (Supplemental Table 1). Quantile-quantile (Q-Q) plots were generated in R v3.2.4 using the “qqman” package^18^ for genomic control adjusted p-values from the meta-analysis. Manhattan plots were generated in R v3.2.4 and Integrated Genome Viewer v2.3 (https://www.broadinstitute.org/software/igv/home).^17^ Forest plots were generated using OpenMetaAnalyst (http://www.cebm.brown.edu/open_meta/index.html).

Further bioinformatics investigation of the top genome-wide significant locus from the meta-analysis was conducted. SNPs linked to the lead SNP were determined by examining the LD block structure using Plink and LocusZoom, and SNP information was retrieved from the Manhattan plot in IGV2. For further analysis, we selected 9 linked SNPs with R^2^>0.6, within 0.5 Mb of the lead SNP, and that showed nominal association (p<1x10^-5^) in our results. We used a cutoff of R2>0.6 because we wished to identify all SNPs that could possibly affect the activity of nearby genes and also account for the association of rs71404070 with rotator cuff injury The genomic context of each SNP was investigated using the 1000 Genomes Browser (http://browser.1000genomes.org/index.html), UCSC Genome Browser (https://genome.ucsc.edu/), and RegulomeDB (<http://regulomedb.org/>)^2^ web tools. Whether each SNP is an expression quantitative trait locus (eQTL) was queried using the NCBI eQTL Browser (http://www.ncbi.nlm.nih.gov/projects/gap/eqtl/index.cgi) and the Genotype-Tissue Expression (GTEx) Portal (http://www.gtexportal.org/home/).

References.

**1.** Banda Y, Kvale MN, Hoffmann TJ, et al. Characterizing Race/Ethnicity and Genetic Ancestry for 100,000 Subjects in the Genetic Epidemiology Research on Adult Health and Aging (GERA) Cohort. *Genetics.* 2015;200(4):1285-1295.

**2.** Boyle AP, Hong EL, Hariharan M, et al. Annotation of functional variation in personal genomes using RegulomeDB. *Genome research.* 2012;22(9):1790-1797.

**3.** Cavalli-Sforza LL. The Human Genome Diversity Project: past, present and future. *Nat Rev Genet.* 2005;6(4):333-340.

**4.** Graffelman J, Moreno V. The mid p-value in exact tests for Hardy-Weinberg equilibrium. *Stat Appl Genet Mol Biol.* 2013;12(4):433-448.

**5.** Hoffmann TJ, Kvale MN, Hesselson SE, et al. Next generation genome-wide association tool: design and coverage of a high-throughput European-optimized SNP array. *Genomics.* 2011;98(2):79-89.

**6.** Hoffmann TJ, Zhan Y, Kvale MN, et al. Design and coverage of high throughput genotyping arrays optimized for individuals of East Asian, African American, and Latino race/ethnicity using imputation and a novel hybrid SNP selection algorithm. *Genomics.* 2011;98(6):422-430.

**7.** Howie B, Fuchsberger C, Stephens M, Marchini J, Abecasis GR. Fast and accurate genotype imputation in genome-wide association studies through pre-phasing. *Nat Genet.* 2012;44(8):955-959.

**8.** Howie B, Marchini J, Stephens M. Genotype imputation with thousands of genomes. *G3 (Bethesda).* 2011;1(6):457-470.

**9.** Howie BN, Donnelly P, Marchini J. A flexible and accurate genotype imputation method for the next generation of genome-wide association studies. *PLoS Genet.* 2009;5(6):e1000529.

**10.** Jorgenson E, Makki N, Shen L, et al. A genome-wide association study identifies four novel susceptibility loci underlying inguinal hernia. *Nat Commun.* 2015;6:10130.

**11.** Kvale MN, Hesselson S, Hoffmann TJ, et al. Genotyping Informatics and Quality Control for 100,000 Subjects in the Genetic Epidemiology Research on Adult Health and Aging (GERA) Cohort. *Genetics.* 2015;200(4):1051-1060.

**12.** Li JZ, Absher DM, Tang H, et al. Worldwide human relationships inferred from genome-wide patterns of variation. *Science.* 2008;319(5866):1100-1104.

**13.** Manichaikul A, Mychaleckyj JC, Rich SS, Daly K, Sale M, Chen W-M. Robust relationship inference in genome-wide association studies. *Bioinformatics (Oxford, England).* 2010;26(22):2867-2873.

**14.** Marchini J, Howie B. Genotype imputation for genome-wide association studies. *Nat Rev Genet.* 2010;11(7):499-511.

**15.** Patterson N, Price AL, Reich D. Population structure and eigenanalysis. *PLoS Genet.* 2006;2(12):e190.

**16.** R. *Team. R: A language and environment for statistical computing*; 2013.

**17.** Robinson JT, Thorvaldsdottir H, Winckler W, et al. Integrative genomics viewer. *Nat Biotechnol.* 2011;29(1):24-26.

**18.** Turner SD. qqman: an R package for visualizing GWAS results using Q-Q and manhattan plots. *biorXiv* 2014.
